# 基于超高效液相色谱-质谱法的肽段分析中非特异性吸附评估及通用型最小化策略

**DOI:** 10.3724/SP.J.1123.2021.12012

**Published:** 2022-07-08

**Authors:** Ying ZHANG, Jing YANG, Yuexin MA, Ling CAO, Qing HUANG

**Affiliations:** 1.南京中医药大学药学院, 江苏 南京 210023; 1. College of Pharmacy, Nanjing University of Chinese Medicine, Nanjing 210023, China; 2.江苏省食品药品监督检验研究院, 国家药品监督管理局化学药杂质谱研究重点实验室, 江苏 南京 210019; 2. Jiangsu Institute for Food and Drug Control, National Medical Products Administration, Key Laboratory for Impurity Profile of Chemical Drugs, Nanjing 210019, China

**Keywords:** 超高效液相色谱-质谱, 蛋白质组学, 非特异性吸附, 定量, 残留, 多肽, ultra-performance liquid chromatography-mass spectrometry (UPLC-MS), proteomics, nonspecific adsorption, quantification, carryover, peptides

## Abstract

蛋白质组学技术在多肽和蛋白质类新型治疗药物的开发、临床诊断生物标志物的深入发掘中应用广泛。然而,多肽和蛋白质类大分子的非特异性吸附性质给分析方法的开发带来极大挑战,亟须一种通用型的策略去评估和降低非特异吸附对超高效液相色谱-质谱(UPLC-MS)大分子检测造成的负面影响。研究以牛血清白蛋白(BSA)为模型,探讨其酶解后多肽组理化性质与吸附程度之间的相关性;根据肽段的响应和吸附程度设计分级策略;针对高响应、强吸附的Class Ⅱ类肽段,从样品制备中离心管、进样瓶的选择,乃至液相色谱系统中色谱柱固定相、流速、梯度、柱温、洗针液的选择全过程设计试验,探讨非特异吸附的影响因素及其通用型最小化策略。结果显示,肽段的被吸附程度与其理化参数HPLC指数(HPLC Index)、肽段长度等显著相关(*p*<0.05),但仅凭上述参数仅能解释30%肽段的被吸附程度。改性的聚丙烯材料可使肽段溶液在储存或前处理过程中获得较高的回收率(24 h内回收率大于80%)。在对液相色谱条件的考察和优化过程中发现,C_8_填料的色谱柱、高流速、缓梯度以及强洗针液,可使残留量降至最低(降低为原来的1/150)。柱温对残留的影响在肽段间存在较大个体差异,需要对不同的肽段具体分析以得到较少量的残留。研究以详实的数据考察并最小化模型肽段组在分析过程中的非特异吸附,提示了蛋白质类大分子药物分析方法建立中应重点关注的影响因素及其有效的解决方案。

## Abstract (en)

Proteomics technology is being increasingly used in the development of novel therapeutic peptides and protein drugs, and also in the intensive search for clinical biomacromolecule diagnostic biomarkers. Ultra-performance liquid chromatography-mass spectrometry (UPLC-MS) is a rapid method to analyze peptides and proteins in low abundance. However, the nonspecific adsorption properties of peptides may lead to the loss or interference of the analytes throughout the analytical process. This unique nonspecific adsorption property is the main reason for the false negative and false positive results obtained through quantification, as well as for the poor precision, accuracy, linear range, and sensitivity, all of which impose significant challenges in the development of analytical methods.

Accordingly, a general strategy was established to evaluate and reduce the negative impact of the nonspecific adsorption of peptides on UPLC-MS analysis. In this study, bovine serum albumin (BSA) was used as a model protein to explore the correlation between the physicochemical properties of 50 peptides obtained by the enzymatic digestion of BSA, as well as the degree of nonspecific adsorption. First, these peptides were classified into four categories according to their response and the degree of adsorption in the pretreatment containers and LC system. Next, the factors influencing the adsorption of 12 Class Ⅱ peptides, which were highly responsive and susceptible to adsorption, were systematically studied in terms of several aspects, including: (1) time-dependent adsorption on centrifuge tubes of three kinds (Protein-LoBind polypropylene tube and two types of polypropylene tubes); (2) time-dependent adsorption on sample vials of three kinds (Protein-LoBind polypropylene vial, polypropylene vial, and glass vial); (3) carryovers on chromatographic columns with six different stationary phases (Polar C_18_, Cortecs C_18_^+^, PFP, BEH C_18_, CSH C_18_, and BEH C_8_); (4) carryovers at different chromatographic gradients (2%B-30%B, 2%B-40%B, 2%B-50%B, and 2%B-60%B within 3 min), flow rates (0.2, 0.3, and 0.4 mL/min), and column temperatures (30, 40, 50, and 60 ℃); and (5) carryovers using different washing needle solutions (0.2% formic acid in 10% acetonitrile and 0.2% formic acid in 90% acetonitrile).

The results showed that parameters such as the HPLC index and amino acid length of peptides were significantly correlated with their degree of adsorption (*p*<0.05), However, the above parameters can only explain the adsorption degree of 30% of the peptides. The use of the modified polypropylene material resulted in higher recovery (recovery rate>80% within 24 h) of the peptide solution during storage or pretreatment. During protein/peptide pretreatment and storage, good overall recoveries (recovery rate>80% within 24 h) were obtained using centrifuge tubes and sample vials made of the modified polypropylene material. Analysis and optimization of the LC conditions revealed that the use of the C_8_ chromatographic column, a high flow rate (0.4 mL/min), slow gradient (2%B-50%B within 3 min), and strong needle solution (0.2% formic acid in 90% acetonitrile) could minimize the carryover. However, the effect of the column temperature on the carryover varied considerably from peptide to peptide, and hence, requires further analysis for specific peptides. The combined optimization of the above experimental conditions resulted in minimal (approximately 1/150) or no adsorption of the Class Ⅱ peptides that were susceptible to adsorption in the analytical process.

In this study, a workflow was designed to standardize the procedures for evaluating and reducing peptide adsorption. Detailed data were collected to elucidate the key risk factors and corresponding general mechanism of nonspecific adsorption throughout the analysis. Thus, this study serves as a reference for the development of analytical methods for peptides and proteins with different physicochemical properties. In future work, the risk factors should be assessed during the development and validation of protein-based macromolecular analysis methods. In conclusion, it is important to implement adequate and appropriate measures to ensure risk elimination or minimization.

近年来,蛋白质组学技术在肽和蛋白质类新型治疗药物的蓬勃发展以及临床新型大分子生物标志物的深入发掘中被日益广泛应用^[1,2]^。应用方式的迭代对生物大分子的分析技术提出了更高的要求^[3]^。基于蛋白质特征肽段检测的自下而上的蛋白质组学技术(bottom up proteomics)是现有研究中具有较高灵敏度与分辨率的蛋白质定性定量方法^[4,5]^。开发多肽的生物分析方法是极具挑战的,除了所需的低检出限外,多肽的非特异性吸附性质,使其极易在接触到的材料表面发生吸附,进而导致分析全流程中待测物的丢失或干扰,给定性和定量分析引入巨大风险^[6,7]^。例如在蛋白组学研究的质谱数据库搜索中,即使系统中微量肽段的损失或残留亦可能导致假阳性或假阴性结果^[8]^。而在高灵敏度的多肽定量方法的开发中,肽段的非特异吸附对定量分析的线性、准确度和精密度均有负面影响^[9]^。低浓度肽段溶液的吸附性质会更加明显,表现形式为标准曲线的非线性,最终导致定量限的不必要升高以及方法的重复性差^[10]^。

已有一些研究在分子水平上解释这种吸附行为,然而目前对其潜在的机制和相互作用仍然知之甚少。Eeltink等^[11]^基于分子动力学模拟,提出了一种三相分子机制解释肽段从溶液吸附到强相互作用不带电固定相上的原理。Kristensen等^[12]^研究了样品容器对阳离子多肽吸附的影响,当1 μmoL/L肽溶液在硼硅酸盐或聚丙烯瓶中存储1 h后,肽段的回收率仅有10%~20%。也有研究通过在溶剂中添加有机试剂、酸/碱性溶液、表面活性剂、吸附竞争剂或调整流动相组成等方法减少这类吸附^[13,14]^。这些研究论文大多对一组特定的多肽和/或表面材料进行研究,但均未给出可用来预测多肽吸附特性的规律,也未给出通用的解决吸附的方法^[15]^。

鉴于上述问题,本研究选择牛血清白蛋白(BSA)作为模型蛋白质,以其酶解后的肽段作为包含亲水性和疏水性多肽的“典型”多肽组样本。首先通过超高效液相色谱-高分辨质谱(UPLC-HRMS)的测定,分析常见多肽理化参数与上述多肽组的非特异吸附程度的关联性。然后基于超高效液相色谱-三重四极杆质谱(UPLC-QQQ-MS/MS)建立对强吸附肽段吸附程度的评估方法,从样品制备至分析测定建立全过程试验设计,考察不同材质的制备、储存耗材对肽段吸附的影响,以及考察不同色谱条件对肽段残留的影响,最终提出多肽全流程分析中减少非特异性吸附的通用型策略。

## 1 实验部分

### 1.1 仪器、试剂与材料

BSA标准品(纯度≥96%)、二硫苏糖醇、碘代乙酰胺、亮氨酸脑啡肽均购自美国Sigma-Aldrich公司。胰蛋白酶购自美国Progema公司;碳酸氢铵和乙腈购自德国Merck公司;甲酸购自美国Thermo Fisher公司;所有实验用水均为超纯水(Millipore,美国)。

数据采集与分析:超高效液相色谱-高分辨飞行时间质谱联用仪(Synapt XS, Waters,美国);超高效液相色谱-三重四极杆质谱仪(6500 plus, AB Sciex,美国); Unifi操作软件(Waters,美国)。

电荷性、酸碱性、水溶性、相对分子质量的理论数值参考网站:https://www.genscript.com.cn/tools/peptide-molecular-weight-calculator; HPLC指数^[16]^、等电点(pI)的理论数值经Unifi软件计算得到;平均疏水指数(grand average of hydropathicity, GRAVY)参考网站:https://web.expasy.org/protparam/。数据分析以SPSS Statistics 23软件进行。

### 1.2 样品制备方法

取10 mg BSA溶于10 mL水中,制得1 mg/mL蛋白储备液,进一步以水稀释为100 μg/mL的工作液。取200 μL上述工作液于蛋白质低吸附离心管中;加入65 μL 500 mmol/L碳酸氢铵和60 μL 50 mmol/L二硫苏糖醇,于60 ℃水浴加热60 min对蛋白质进行还原;放冷至室温后加入120 μL 50 mmol/L碘代乙酰胺,于暗处反应30 min进行烷基化;加入100 μg/mL的胰蛋白酶5 μL,于37 ℃水浴中酶解8 h,加入甲酸20 μL终止反应,12000 g离心15 min后,取200 μL上清置于蛋白质低吸附的进样瓶中作为混合肽段溶液待测。

### 1.3 超高效液相色谱-高分辨质谱方法参数

色谱条件:色谱柱采用Waters Acquity Premier Peptide CSH C_18_(100 mm×2.1 mm, 1.7 μm);柱温为40 ℃;流速为0.25 mL/min;流动相A、B两相分别为0.1%甲酸水溶液和0.1%甲酸乙腈溶液。洗脱梯度为0~1 min, 1%B; 1~13 min, 1%B~40%B; 13~13.1 min, 40%B~90%B; 13.1~16 min, 90%B; 16~16.1 min, 90%B~1%B; 16.1~20 min, 1%B。进样器温度10 ℃;进样量5 μL。

质谱条件:毛细管电压3 kV,锥孔电压30 V,离子源温度120 ℃,脱溶剂气温度450 ℃,锥孔气流速25 L/h,脱溶剂气流速800 L/h。电喷雾电离(ESI)源、正离子模式下测定,MS^E^模式采集,扫描范围 *m/z* 50~2000;数据采集时使用亮氨酸脑啡肽校正液进行实时质量校正,以保证采集质量数的准确性与重复性。采集后的数据使用Unifi软件处理。

### 1.4 相对残留量的测定和肽段分级策略

将1.2节混合肽段溶液经上述条件采集、Unifi软件分析后,可得BSA酶解后肽段组的实际肽段组成和每个肽段的响应值Area_(供试品溶液)_。

在进样上述供试品溶液后连续进样3针空白溶剂,以3针空白溶剂中检测到的对应肽段响应之和Area_(Blank 1+Blank 2+Blank 3)_计为该肽段的残留总量,该肽段的相对残留量为肽段的残留总量与肽段响应值的比值。

基于肽段的响应与相对残留量,可将BSA酶解后的肽段组分为如下四类:Class Ⅰ,响应高且无残留的肽段;Class Ⅱ,响应高但有残留的肽段;Class Ⅲ、Class Ⅳ分别为响应低,无吸附和有吸附的肽段。响应的高低以是否大于中位数计,有无残留以Area_(Blank 1+Blank 2+Blank 3)_是否有检出判断。

### 1.5 超高效液相色谱-三重四极杆质谱方法参数

色谱条件:色谱柱采用Waters ACQUITY UPLC BEH C_8_(100 mm×2.1 mm, 1.7 μm);柱温30 ℃;流速0.4 mL/min;流动相A、B两相分别为0.2%甲酸水溶液和0.2%甲酸乙腈溶液。洗脱梯度为0~2 min, 2%B; 2~5 min, 2%B~60%B; 5~5.1 min, 60%B~90%B; 5.1~8 min, 90%B; 8~8.1 min, 90%B~2%B; 8.1~11 min, 2%B。进样器温度10 ℃;进样量5 μL。洗针液为90%乙腈水溶液(含0.2%甲酸)。

质谱条件:离子化电压5500 V;气帘气压力0.14 MPa;离子源温度500 ℃;喷雾气、辅助加热气压力0.38 MPa。ESI源正离子模式下测定,多反应监测(MRM)模式采集,12条Class Ⅱ类肽段的离子对、碰撞能量(CE)、去簇电压(DP)值经Skyline软件协助优化后结果如表1所示。

**表 1:** BSA酶解后Class Ⅱ类肽段的离子对信息及MRM参数

Number	Peptide	[M+H]^+^	Q1 (m/z)	Q3 (m/z)	Declustering potential/V	Collision energy/eV
P1	DAFLGSFLYEYSR	1567.743	784.38	1121.53	49.9	35.4
P2	GLVLIAFSQYLQQCPFDEHVK	2492.264	831.43	383.24	47.8	35.9
P3	ECCHGDLLECADDR	1749.662	583.89	765.28	58.7	28.0
P4	TVMENFVAFVDK	1399.693	700.35	1199.58	53.6	29.3
P5	LFTFHADICTLPDTEK	1907.921	636.65	589.28	56.4	30.6
P6	DDPHACYSTVFDK	1554.653	518.89	609.32	61.6	24.9
P7	MPCTEDYLSLILNR	1724.835	862.92	715.45	46.5	47.3
P8	LVNELTEFAK	1163.631	582.32	951.48	58.8	27.5
P9	SLHTLFGDELCK	1419.694	473.90	721.32	63.6	22.7
P10	VPQVSTPTLVEVSR	1511.843	756.43	900.52	51.1	42.1
P11	HLVDEPQNLIK	1305.716	653.36	1055.57	55.7	35.0
P12	LGEYGFQNALIVR	1479.795	740.40	1017.58	51.8	43.3

## 2 结果与讨论

### 2.1 肽段的理化性质与吸附程度

BSA是牛血清中的球蛋白,包含607个氨基酸残基(为66.446 kDa)。本研究以BSA为模型蛋白质,将其用胰蛋白酶酶解,酶解后的肽段作为包含亲水性和疏水性多肽的典型性多肽组样本。经1.3节方法数据采集和分析,结果显示,BSA酶解后共测定到长度5~28个氨基酸的肽段共计50条。将这些肽段的常见理化参数(包括电荷性、酸碱性、水溶性、肽段长度、GRAVY、HPLC指数、pI值和亲水性残基占比)与肽段在液相色谱系统吸附程度(相对残留量)及离心管中的吸附程度(肽段溶液在聚丙烯材质的离心管中放置24 h后的回收率)进行相关性分析。肽段溶液在液相色谱系统中的相对残留量与肽段的HPLC指数、水溶性和肽段长度呈显著相关(*p*<0.05);在离心管中的回收率与HPLC指数和肽段长度呈极显著相关(*p*<0.01)。但仅有HPLC指数这一参数在理化参数和液相色谱系统中吸附和离心管中吸附的多元线性回归分析中均呈显著相关(*p*<0.05),相关系数(*R*^2^)分别为0.223、0.268。

上述结果提示我们,肽段的HPLC指数、水溶性和肽段长度在筛选定量用特征肽段阶段虽可用于初步评估肽段的吸附性质,但对肽段吸附程度差异的解释度仍不足,无法用于准确预测肽段的吸附情况。研究人员需要根据试验结果评估肽段组实际吸附情况、探索减小前处理和分析过程中非特异吸附的方案。

### 2.2 肽段的分类策略

在无法仅使用理化参数对肽段吸附性质进行准确预测的情况下,筛选用于定量的特征肽段时,需要通过具体实验对备选肽段的吸附性质和质谱响应进行预筛选。本研究根据BSA酶解后肽段组的质谱响应值、残留程度设计了肽段的四分类策略。以BSA实测得的50条酶解肽段为例,分级为Class Ⅰ类高响应、无吸附肽段11条;Class Ⅱ类高响应但有吸附肽段12条(如图1a所示)。

**图 1:**
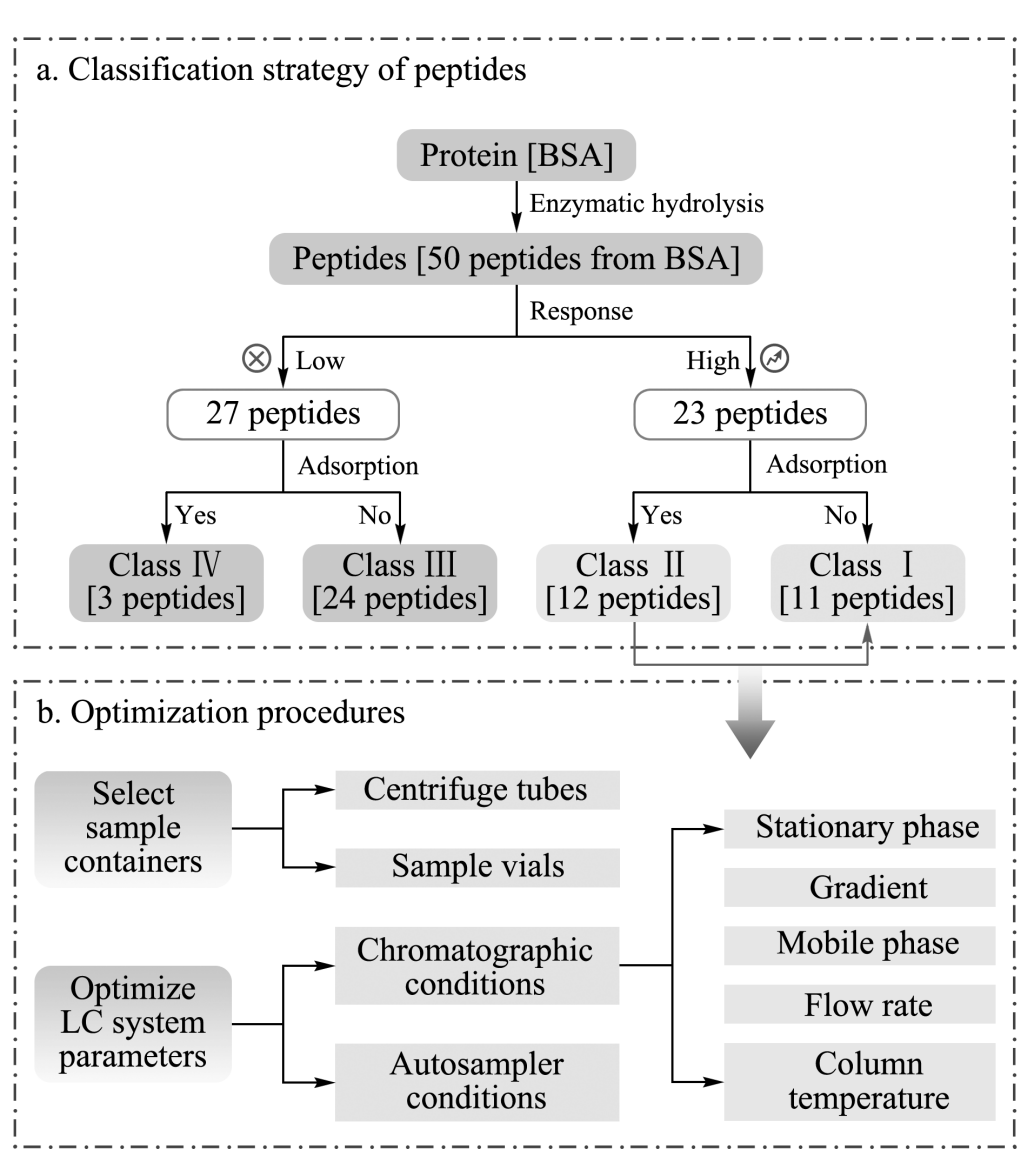
(a)肽段的分级策略及(b)非特异性吸附因素 考察和优化流程

Class Ⅰ类是性质最优异的备选待测肽段,但在实际研究中,初始Class Ⅰ类的肽段数量可能非常有限,且需要进一步结合肽段的特异性、修饰情况、酶解性质等进行逐级漏斗式筛除,最终甚至无法直接从中筛得可用的目标肽段。因此,通过实验条件的优化使肽段最少吸附甚至不吸附,将Class Ⅱ类肽段转化为Class Ⅰ类,可极大地增加蛋白/多肽分析方法开发的灵活性。

鉴于上述目的,本研究针对方法开发全过程中可能产生非特异吸附的影响因素设计试验(图1b所示),从前处理装置的选择与色谱条件优化两个角度,尝试减小或消除BSA酶解后Class Ⅱ类肽段的吸附。

### 2.3 前处理装置对肽段回收率的影响与选用

#### 2.3.1 离心管对回收率的影响

将BSA酶解后的混合肽段溶液(1.2节)稀释至以总蛋白计5000 (高)、500(中)、50(低)ng/mL 3个水平的样品溶液,分别取800 μL置于3种1.5 mL离心管中(*n*=3)。离心管种类分别为改性聚丙烯材质(PP-LB)的蛋白质低吸附离心管和实验室常用的普通聚丙烯材质(PP-A、PP-B)离心管。为模拟酶解环境,将装有3个水平样品溶液的上述离心管置于37 ℃水浴中,分别在0、5、12、24 h后取出,涡旋混匀后取100 μL溶液至PP-LB进样瓶中,以1.5节方法测定,回收率为第*x* h测定的峰面积与第0 h测定峰面积的比值。

结果显示:3种不同离心管的平均回收率在中、高水平下均无显著差异(*p*=0.51,回收率均大于80%);在低水平下PP-LB管的平均回收率(94.01%)显著高于低水平下PP-A(82.43%)和PP-B(76.62%)管。此外,PP-LB离心管在所有水平下,对12条典型有吸附肽段的回收率离散程度(SD)、最小回收率(Min)、回收率小于80%的肽段个数(Count), 3项指标亦均优于PP-A、PP-B管(见图2a)。离心管的材质一般为普通聚丙烯,肽段上若有疏水性残基则易与其发生疏水性结合,这种结合不会因振荡等外力而脱落,易造成肽段的损失。本实验结果表明,经过改性处理的蛋白质低吸附的聚丙烯材质离心管表现出良好的普适性的抗吸附性能。有研究人员通过X射线光电子能谱(XPS)分析表明,改性材料表面的氧含量高于普通的聚丙烯管,较高的含氧量可能会增加管壁的水合程度,从而抑制与肽段的结合^[17]^。因此,对肽段不易吸附的改性聚丙烯离心管是样品配制、储存、酶解反应容器等步骤中的优选。

**图 2:**
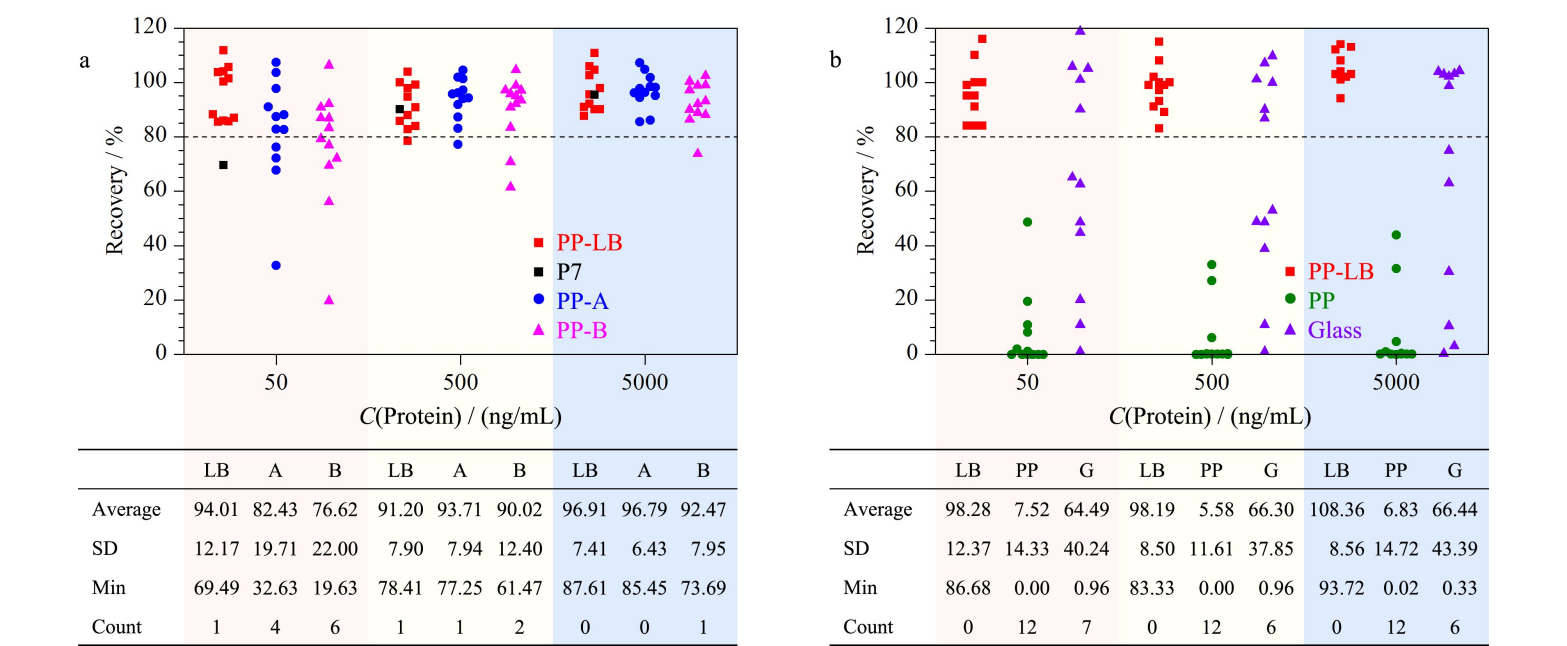
肽段溶液在不同样品容器中放置24 h后的回收率

此外,我们还观察到,肽段组在低浓度溶液中发生吸附的相对损失显著高于中、高浓度样品溶液。以图2a中典型肽段P7为例,在低、中、高浓度溶液下的回收率分别为69.49%、89.98%、95.40%。这可能归因于湿固体表面积的结合能力有限^[17]^,即吸附能力有限,这一现象会体现为肽段标准曲线的非线性。鉴于低浓度的肽段更容易被吸附这一特性,配制尽可能高浓度的储备液或在低浓度下合理添加抗吸附剂可提高实验准确性。

最后,大部分肽段的被吸附情况都与时间长度呈正相关,但吸附速率呈现个体间差异。仍以肽段P7为例,其回收率在3种不同离心管中随时间变化情况如图3a所示。5 h内P7在3种离心管中的回收率均大于80%;但随着放置时间的增长,5~24 h内回收率逐渐下降。因此,在研究中通过酶解条件等前处理方法的优化,减少前处理总时长,降低因反应时长导致的肽段损失是十分必要的。

**图 3:**
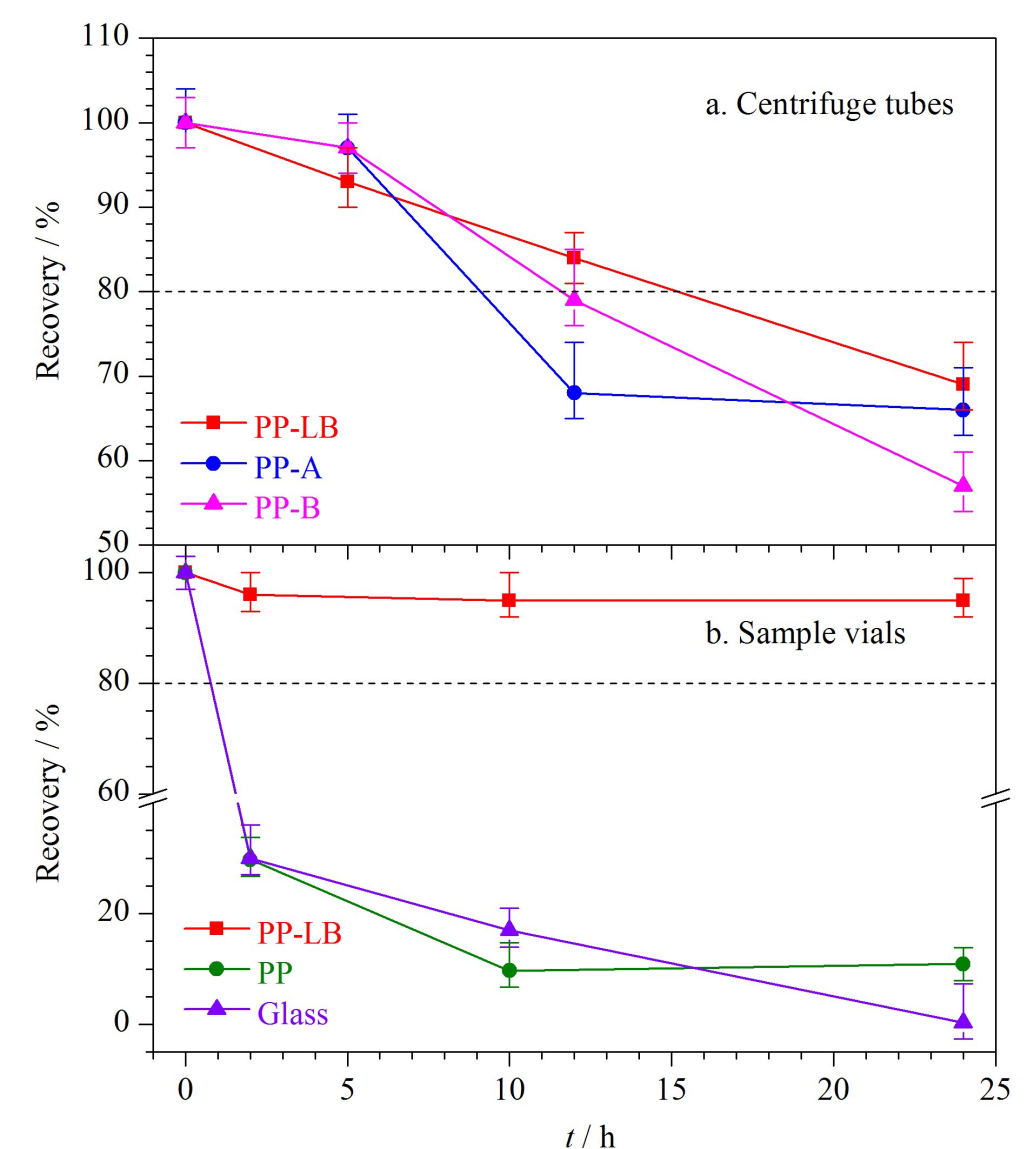
不同样品容器中低浓度肽段P7溶液的回收率变化趋势(*n*=3)

#### 2.3.2 进样瓶对回收率的影响

待测肽段溶液转入进样瓶后,一般放置一定时长后注入UPLC-QQQ-MS/MS测定,因此进样瓶中的吸附特征考察亦十分必要。本研究考察了蛋白质低吸附聚丙烯、聚丙烯、玻璃3种常见材质进样小瓶对肽段吸附程度的影响。分别取2.3.1节高、中、低3种水平的样品溶液200 μL于3种不同材质的进样小瓶中(*n*=3)。将装有肽段溶液的进样小瓶置于进样盘(10 ℃),于0、2、10、24 h后进样分析,计算回收率。结果如图2b所示:(1)在蛋白质低吸附聚丙烯进样瓶中,所有12条肽段均可稳定保存24 h(回收率大于80%);回收率的离散程度、最小回收率、回收率小于80%的肽段个数,3项指标亦明显优于玻璃瓶和聚丙烯瓶;(2)在低浓度的情况下,玻璃瓶中仅有5条肽段(P3、P6、P8、P9、P11)的回收率高于80%,其余7条肽段回收率分散分布在0~70%之间。玻璃材质中的肽段吸附呈现较大的个体差异性,这可能与不同肽段残基带电性质与玻璃表面硅烷醇基团的静电吸附作用不同有关^[11]^; (3)未改性的聚丙烯瓶中所有肽段回收率均低于80%,大部分肽段被完全吸附(回收率<10%),故不建议使用。

肽段在进样瓶中的吸附现象与离心管(2.3.1节)中不同的是:(1)上述吸附现象未随肽段溶液浓度增大而有所改善(见图2),在本实验测试的3个浓度下未见明显的吸附饱和;(2)肽段溶液在进样小瓶中被吸附过程非常迅速,大量的肽段快速吸附到玻璃小瓶和塑料小瓶壁上进而导致回收率较低。仍以P7为例,其在3种不同进样瓶中的回收率随时间变化的趋势如图3b所示。蛋白质低吸附进样瓶中肽段可在24 h内不被吸附,但玻璃和普通聚丙烯材质的进样瓶中肽段组在2 h内即被迅速吸附;在2~24 h吸附速率放缓但仍持续发生,甚至可被吸附完全。在测定含有低浓度蛋白质的样品时,大量吸附可造成假阴性的检测结果。因此,由于无法保证进样的即刻性和样品在进样器中放置时间的一致性,选择蛋白质低吸附的进样瓶开展实验是尤为重要的。

### 2.4 液相色谱系统中肽段残留的影响与优化

#### 2.4.1 色谱条件对残留影响的考察

2.3节证明了肽段溶液在前处理接触材料表面上的吸附会导致分析中回收率低、重复性差等问题,而肽段溶液在液相色谱系统中发生吸附,则会造成样品间的交叉污染、假阳性、准确性和重复性差、线性范围窄等一系列问题^[18]^。为此,本部分对5个常见色谱条件,即色谱柱固定相类型、柱温,流动相的流速、梯度、甲酸的体积分数对色谱柱上的肽段残留的影响进行了系统考察。初始色谱条件为:柱温40 ℃,流速0.3 mL/min,流动相A、B两相分别为0.2%甲酸水溶液和0.2%甲酸乙腈溶液。下述色谱条件优化过程中条件在此基础上单一变化。在液相色谱系统中残留的评价标准为:将1.2节制备的混合肽段溶液稀释至10 μg/mL,以如图4a所示的流动相梯度洗脱,包括有效梯度(2~6 min, 2%B~40%B)和高/低有机相交替循环的冲洗梯度来考察色谱柱上残留。每个肽段在色谱柱上的残留量以循环梯度洗脱出的肽段总峰面积计(见图4b)。肽段在色谱柱上的相对残留量的计算方法为高/低有机相交替循环的冲洗梯度检出的峰面积与有效梯度检出峰面积的比值。

**图 4:**
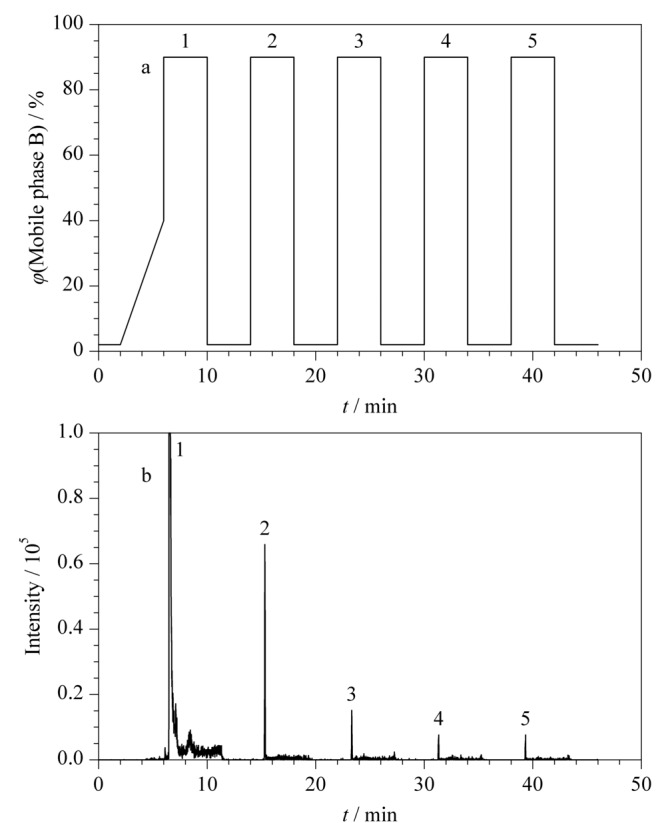
(a)用于评估色谱柱上残留量的梯度洗脱程序及(b)残留肽段的典型色谱图

肽段在色谱柱上的残留受到多种因素的影响,我们首先考察了6种不同的色谱柱固定相(具体参数如表2所示)对肽段残留的影响。结果如图5所示,12条肽段的相对残留量之和在色谱柱上体现为Polar C_18_>PFP>Cortecs C_18_^+^>BEH C_18_>CSH C_18_>BEH C_8_,相对残留量最大和最小值之间相差38倍。C_8_色谱柱对所有肽段都有较低残留的特性,CSH C_18_次之。与小分子相比,肽和蛋白质的保留机制是一个较为复杂的过程,并且受到固定相多种相互作用力的影响^[13]^。推测C_8_色谱柱较短的碳链降低了键合相非极性作用的面积,因此较C_18_保留更弱,残留也较低;性能次优的CSH C_18_柱,在填料表面带电杂化同时,采用高性能表面(HPS)技术,这种有机/无机杂化表面技术能在样品与不锈钢色谱柱之间形成屏障表层,减少接触表面与待测物质的相互作用,使残留降低。

**表 2:** 色谱柱参数

Stationary phase	Pore size/nm	Particle size/μm	Diameter/ mm	Length/ mm
Polar C_18_	10	2.6	2.1	100
Cortecs C_18_^+^	9	1.6	2.1	100
PFP	10	1.7	2.1	100
BEH C_18_	30	1.7	2.1	100
CSH C_18_	13	1.7	2.1	100
BEH C_8_	13	1.7	2.1	100

**图 5:**
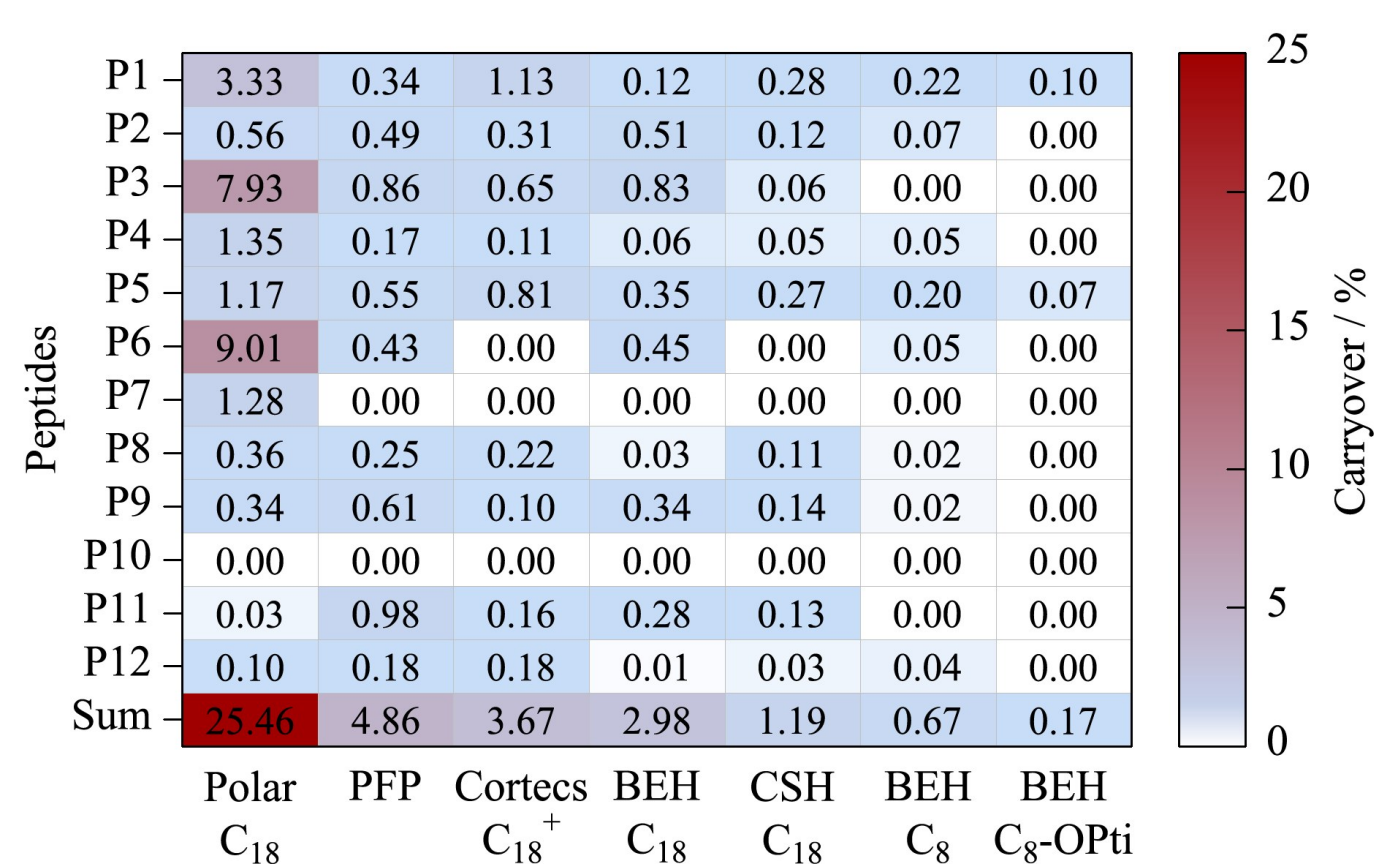
固定相类型对肽段在色谱柱上残留的影响

选择残留量中等的Cortecs C_18_^+^柱,进一步考察流动相中的甲酸体积分数(0.1%、0.2%、0.3%),流速(0.2、0.3、0.4 mL/min),柱温(30、40、50、60 ℃),梯度斜率G1、G2、G3、G4(2~6 min: 2%B~30%B、2%B~40%B、2%B~50%B、2%B~60%B)对肽段在色谱柱上残留的影响。结果显示,流速、梯度及甲酸的体积分数对肽段残留的影响,在12条肽段上呈现一定程度的同趋势变化,因此以12条肽段的相对残留量均值表征不同条件对残留的影响(如图6)。(1)在0.2、0.3、0.4 mL/min的流速下,肽段残留量随流速的增加而显著降低(见图6a)。这可能是由于流速增大使柱压增加,进而提高肽段在色谱柱头的溶解度,使其在柱头处的残留减少,故本实验中0.4 mL/min为优选流速。(2)4个梯度斜率G1、G2、G3、G4对残留量的影响考察结果如图6b所示,随着G1~G4梯度的逐渐减缓,残留量亦随之降低。较缓梯度可以使大体积的肽段分子在临界洗脱的有机溶剂浓度下被冲洗更长时间,从而降低残留^[11]^。但需指出的是,梯度过缓会增加分析时间,所以在研究中可平衡残留量与分析时长、分析通量,根据实验目的选择梯度。(3)流动相中甲酸的体积分数对残留的影响如图6c所示,甲酸的体积分数的增加使残留量降低,这可能与肽段溶解度的增加或偶极相互作用、离子相互作用的降低有关^[19,20]^。但甲酸的体积分数过高(如本例中0.3%较之0.2%)亦会抑制离子化效率,故应权衡实际残留量与灵敏度要求,根据实验目的酌情选择流动相内加入甲酸的百分含量。

柱温对残留的影响较为复杂,呈现出肽段的个体差异(见图6d): 12条肽段在30~60 ℃条件下,残留与柱温呈现正相关(如P8)、负相关(如P1),抑或影响微弱(如P6)3类情况。此结果与Honorine等^[14]^研究论述的高柱温有助于降低残留有所差异。这种肽段个体化的差异在Cortecs C_18_^+^、BEH C_18_和BEH C_8_ 3根色谱柱之间表现亦非完全一致。我们推测这种复杂现象可能是多因素综合作用的体现:其一,为柱温、柱头压力乃至肽段溶解度的综合作用。低柱温下系统压力更高,柱头处肽段溶解度增加而吸附减少^[21]^。其二,柱温与肽段空间结构乃至与固定相相互作用力的综合作用。在低温时,肽段由于分子内基团的相互作用可能发生弯曲和折叠;在中等温度时,随着温度升高肽链展开,与固定相相互作用的位点增多;在高温下完全展开时,表现为趋近小分子,即随着温度升高保留量(残留)有所下降;故在低、中、高整个温度范围内呈现出二阶抛物线^[19]^。上述因素随不同肽段氨基酸组成不同、固定相种类不同而呈现出差异化的结果,故对于柱温的选择应随待测肽段的不同做具体分析。本研究中最终使用的是残留量最低的色谱柱BEH C_8_,在此色谱柱上有3条肽段不残留,6条肽段随柱温升高残留量略增,3条肽段随柱温升高,残留量降低,因此选择30 ℃作为本研究选择的柱温。

**图 6:**
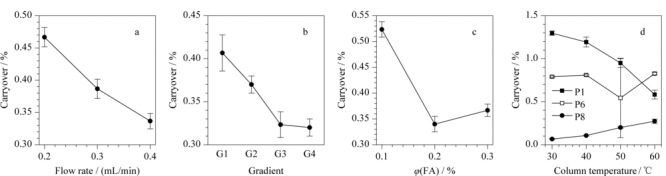
不同色谱条件对肽段溶液在色谱柱上肽段残留量的影响(*n*=3)

综上所述,本研究得出的BEH C_8_色谱柱、0.4 mL/min流速、G4梯度、30 ℃柱温、0.2%甲酸的流动相作为减少肽段组残留的最佳色谱条件。结果如图5中BEH C_8_-Opti,将BEH C_8_色谱柱上的总体残留由6种色谱柱中最低的0.67%,进一步降低至0.17%;原8条有残留的肽段中,6条降至零残留,色谱柱上的总残留量从25.46%降至0.17%(150倍)。此研究极大地拓展了定量肽段可选择的空间。

#### 2.4.2 洗针液对残留影响的考察

最后,我们考察了“弱”洗针液10%乙腈水溶液(含0.2%甲酸)、“强”洗针液90%乙腈水溶液(含0.2%甲酸)下,肽段在进样针及管路中的残留情况。结果显示,在排除柱上残留因素后,使用弱洗针液时,12条肽段的平均相对残留量约为0.01%,使用强洗针液时肽段在进样针上均无残留。在本实验研究中,针上残留相对柱上残留为次要因素,可通过强洗针液清洗至无残留。

## 3 结论

非特异性吸附是多肽、蛋白质定性研究中假阴性、假阳性以及定量分析中精密度、准确度、线性范围、灵敏度欠佳的主要原因之一^[20]^。本文提出了一种评估和减小肽段组非特异吸附的通用型策略,对于模型蛋白中Class Ⅱ类易吸附的肽段组,通过在标准制备过程中合理选择前处理装置以及在液相色谱分析中优化色谱条件,极大地提高了回收率并将肽段在液相色谱系统中的残留降至最低。

本研究为吸附表象与生物大分子理化性质的深入研究提供了新的思路;设计的优化工作流程可为不同理化性质多肽和蛋白质分析方法开发提供参考;产生的详实数据揭示了分析全流程中的吸附风险点和影响因素,在方法开发和验证过程中,应该明确这些风险的存在,并需要采取措施确保它们被消除或最小化。
